# Severity of hypoxia modulates effect of CPAP on myocardial stress as measured by highly sensitive troponin T

**DOI:** 10.1186/s12931-015-0289-0

**Published:** 2015-10-16

**Authors:** Amir Sharafkhaneh, Jennifer Katigbak, Max Hirshkowitz, Hossein Sharafkhaneh, Saba P. Sharafkhaneh, Christie M. Ballantyne, Biykem Bozkurt, Vijay Nambi

**Affiliations:** Section of Pulmonary, Critical Care and Sleep Medicine, Medical Care Line, Michael E. DeBakey Veterans Affairs Medical Center, MED VA Medical Center, Bldg. 100 (111i), 2002 Holcombe Blvd, Houston, TX 77025 USA; Section of Cardiology, Michael E. DeBakey Veterans Affairs Medical Center, 2002 Holcombe Blvd, Houston, TX 77025 USA; Department of Medicine, One Baylor Plaza, Houston, TX 77030 USA; University of Houston, Main campus, Houston, TX USA; University of Saint Thomas, Houston, TX 77007 USA; Center for Cardiovascular Prevention, Houston Methodist DeBakey Heart and Vascular Center, Houston, TX USA

## Dear editor

Obstructive sleep apnea (OSA) is associated with increased risk for cardio- and cerebrovascular diseases [1, 2]. Animal studies propose intermittent hypoxia and the resulting inappropriate activation of sympathetic nervous system [3, 4] as the major link between OSA and cardio- and cerebrovascular comorbidities. However, the OSA severity level at which continuous positive airway pressure (CPAP) therapy offers cardio- and cerebrovascular benefit (reduction in indices of myocardial stress/injury) is a matter of debate. Sub-clinical injury to the myocardium is considered to be a precursor for the development of incident cardiovascular disease including heart failure [5]. Troponin T measured with a high sensitivity assay (Hs-TnT) has been shown to predict incident heart failure and cardiovascular death [6]. Further, increase or decrease in Hs-TnT over time predicted increased or decreased incidence of heart failure and cardiovascular death [7, 8]. Hs-TnT has also shown to be increased with presence and severity of OSA [9]. Stronger correlations were reported with nadir SpO2 [10]. Roca and colleagues, in a cohort of 1645 subjects free of coronary artery disease and heart failure with a median follow up of 12.4 years, reported that hs-TnT was associated with risk of death or incident heart failure across various categories of OSA after adjusting for 17 potential confounders [11]. Surprisingly, a recent study showed increased Hs-TnT after 12 months of CPAP therapy in patients with OSA. However, the utilization of CPAP was not reported [12]. We report changes in Hs-TnT level as a marker of myocardial stress/injury in a cohort of CPAP compliant patients with OSA.

## Methods

This study was approved by institutional review board of Baylor College of Medicine and Research & Development Committee of Michael E. DeBakey VA Medical Center. All participants signed an informed consent form. We enrolled adult subjects with confirmed OSA (apnea + hypopnea index (AHI) of ≥15 obstructive and/or mixed events/h) using attended polysomnography (PSG). Subjects qualifying for the PSG underwent blood sampling and completed an Epworth Sleepiness Scale (ESS). The participants underwent attended CPAP titration and were placed on therapy. The best pressure was the one associated with the lowest AHI while the patient slept 20 min, or more. After titration, subjects received a CPAP machine and related accessories (Respironics, REMStar Pro) with card reader to monitor the compliance of CPAP and were followed for 6 months. Subjects were seen 2–3 times during the study and CPAP compliance was checked during the visit. CPAP efficacy was rechecked with overnight pulse oximetry at the end of the study. Blood was collected in the morning between 7 and 8 AM in EDTA-containing tubes and kept at 4 °C during processing at baseline and after 6 months of CPAP use. Aprotinin (100 μL containing 0.6 TIU per mL of blood) was added to one of the tubes and the samples were then centrifuged at 3000 rpm for 30 min. Hs-TnT concentrations were measured with a novel high sensitivity assay, Elecsys Troponin T (Roche Diagnostics®), on an automated Cobas e411 analyzer with a limit of measurement of 3 ng/L [13].

## Results

**Fig. 1:**
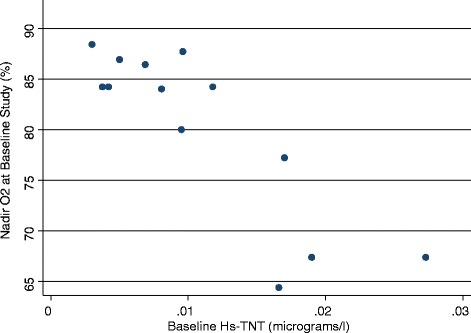
Baseline Hs-TnT correlates strongly with baseline nadir O2 saturation. Y axis presents the nadir SpO2 during the baseline sleep study

**Fig. 2:**
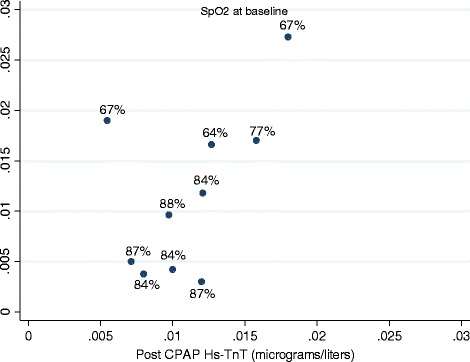
Follow up versus baseline Hs-TnT: The numbers next to each data points show the nadir SpO2 at baseline

Twenty-three subjects were enrolled and 20 subjects completed the study. One subject died unexpectedly at home, from unknown causes. Two subjects were lost to follow up. Baseline and follow samples were available for analysis in 13 and 10 subjects, respectively. The subjects were all male, mean age was 59.7 (SD ± 2) years, body mass index (BMI) 36.5 (SD ± 1.8) kg/m^2^. Baseline polysomnogram showed a total sleep time of 264 min (SD ±121.9), sleep efficiency of 80 % (SD ±10.9 %), latency to sleep onset of 14.3 min (SD ±15.77), wake after sleep onset of 48.8 min (SD ±34.7); with stage N1 of 11 % (SD ±6.5), N2 of 70 % (SD ±14.1), N3 of 11.8 (SD ±10.7) and REM of 11.8 (SD ±10.7). Respiratory parameters at baseline were AHI of 50 (SD ± 6) per hour of sleep, nadir SpO2 of 77 (SD ± 3), and mean time below SpO2 of 90 % of 59.37 min (SD 98.77). Hs-TnT at baseline was elevated and correlated with baseline nadir SpO2 (SpO2) (Correlation Coefficient of 0.889, p <0.001), mean time below SpO2 of 90 % (Correlation of Coefficient of 0.832, p <0.001) but not with AHI or arousal index (Fig. 1). Subjects used CPAP for 165 ± 17 days and 5.3 ± 0.35 h/night. ESS decreased with CPAP therapy (14.6 vs 9.1, *p* < 0.05). Systolic and diastolic blood pressures and heart rate remained unchanged throughout the study period. CPAP effectively reversed hypoxia in all subjects (nadir SpO2 77 ± 3 % at baseline and 89.3 ± 3 post CPAP, *p* = 0.005) (Table 1). Hs-TnT decreased only in subjects with baseline nadir SpO2 of 80 % or lower (*n* = 4) (Fig. 2). The absolute and percent changes in Hs-TnT compared to baseline strongly correlated with baseline SpO2 (Correlation Coefficients of 0.835 and 0.6419 respectively, both p <0.05). The absolute and percent changes in Hs-TnT did not correlate with baseline AHI.

**Table 1:** Sleep and metabolic parameters before and after CPAP use

Pre and Post Treatment Data		
	Baseline	Post-CPAP
AHI (episodes/h)	50 ± 6	-
CPAP pressure (cm H2O)	-	10 ± 3.2
CPAP use (days)	-	165 ± 17
CPAP use (Hrs/day)	-	5.3 ± 0.35
ESS	14.6 ± 1	9.5 ± 1^*^
Nadir SpO2 (%)	77 ± 3	89.3 ± 3^*^
Mean SpO2 (%)	93.2 ± 0.7	93.8 ± 0.62
Systolic blood pressure (mmHg)	124 ± 3	129 ± 4
Diastolic blood pressure (mmHg)	76 ± 2	76 ± 2
Heart rate (bpm)	77 ± 3	72 ± 3
Body weight (Kg)	108 ± 5.3	109.6 ± 5.4^*^
BUN (mg/dl)	14.1 ± 1.19	14.26 ± 1.41
Creatinine (mg/dl)	0.96 ± 0.09	1.0 ± 30.06
Hs-TnT (micrograms/l)	0.012 ± 0.0085	0.011 ± 0.0035^*^

*AHI* Apnea + hypopnea index, *ESS* Epworth sleepiness scale, *Hs-TnT* Highly sensitive troponin T

*: Significant differences compared to baseline (*p* ≤ 0.05) appear in bold

## Discussion

Our study, consistent with others, suggests that Hs-TnT as a biomarker of myocardial stress/injury is elevated in patients with OSA [11]. In contrast, the increased Hs-TnT was associated with severity of hypoxia but not AHI or sleep interruption as measured by arousal index. The CPAP effectively reversed the pathophysiology in the study subjects but only was associated with decline in Hs-TnT in subjects with more severe hypoxia at baseline. Thus, the data suggests that cardio- and cerbro-vascular adverse outcomes related to OSA and the benefits seen with CPAP therapy may greatest to those with pronounced hypoxia as assessed by nadir Sp02 versus other parameters used to assess sleep apnea. A major short coming of our study is the small sample size. While our results are provocative, they must be viewed with caution due to the limited sample size and should be further studied and characterized in larger studies.
